# Maternal and Paternal Identity During the Perinatal Period: A Systematic Review of Conceptualization, Measurement, and Associated Factors

**DOI:** 10.3390/healthcare14213809

**Published:** 2026-11-07

**Authors:** Mercedes Lasarte, Isabel M. Caceres, Alessia Caffieri, Emma Motrico

**Affiliations:** 1 Department of Developmental and Educational Psychology, University of Seville, 41018 Seville, Spain; merlasdel@alum.us.es (M.L.); imcaceres@us.es (I.M.C.); acaffieri@us.es (A.C.); 2 Institute of Biomedicine of Sevilla (IBIS), Av. las Américas, 1, Mairena del Aljarafe, 41927 Sevilla, Spain

**Keywords:** parental identity, maternal identity, paternal identity, perinatal period, pregnancy, postpartum period, parents

## Abstract

**Highlights:**

**What are the main findings?**

- Maternal and paternal identity lack a unified definition, with substantial conceptual and methodological heterogeneity across the available evidence.
- Observational evidence suggests that parental identity dimensions are associated with psychological well-being and parent–fetal attachment, though non-significant and inverse relationships were also identified

**What are the implications of the main findings?**

- This review provides the first structured synthesis of how maternal and paternal identity are conceptualized and measured during the perinatal period, distinguishing direct constructs (internal identity and role attainment) from indirect ones (role salience, event centrality, and identity styles).
- Standardized instruments are essential to improve the comparability and validity of future research.

**Abstract:**

**Background**: The transition to parenthood involves significant psychological challenges, including the development of parental identity. However, research on maternal and paternal identity remains limited and heterogeneous. This systematic review aimed to synthesize empirical evidence on parental identity during the perinatal period, focusing on its conceptualization, assessment methods, and main findings. **Methods**: A systematic review was conducted following PRISMA guidelines. PubMed, PsycINFO, Web of Science, Embase, CINAHL, and Scopus were searched through February 2026. Study selection and data extraction were peer-reviewed, and discrepancies were resolved through discussion. The variables analyzed included study design, sample characteristics, perinatal stage, definitions of identity, assessment methods, the analytical role of identity, and principal findings. Eligible studies were empirical investigations involving mothers, fathers, or both, during pregnancy or the postpartum period. **Results**: A total of 43 full-text articles were assessed for eligibility, of which 15 met the inclusion criteria. The findings revealed substantial conceptual heterogeneity, including direct and indirect measures of parental identity and related constructs such as the maternal role and the transition to parenthood. Parental identity was examined as a dependent, independent, or correlational variable. Parental identity dimensions were associated with psychological well-being, social support, and attachment. However, findings across studies were mixed, with non-significant associations and inverse relationships also observed (e.g., greater pregnancy centrality was associated with elevated posttraumatic stress symptoms). However, the lack of standardized instruments and the methodological variability across studies limited the comparability of their findings. **Conclusions**: Parental identity during the perinatal period is an important but insufficiently standardized construct. Greater methodological rigor and conceptual consensus are needed to advance research and inform interventions in perinatal mental health.

## 1. Introduction

Parental identity development is a dynamic, lifelong process through which individuals construct, negotiate, and refine their self-representation as caregivers [1,2,3]. From psychodynamic and developmental perspectives, identity is defined as a sense of sameness, continuity, and coherence that is perceived by the individual and recognized by others [4]. In this context, parental identity is defined as “the clarity of one’s definition of oneself as a parent” [5] (p. 9). It constitutes a specific domain within global identity and develops dynamically throughout parenthood, with its foundations emerging from early adolescence when parenthood may begin to form part of an individual’s envisioned future self. The Three-Dimensional Model of Identity Formation [6] comprises three identity processes: commitment, in-depth exploration, and reconsideration of commitment. These processes vary according to the timing of parenthood and gender, with women showing greater in-depth exploration than men across all the stages of parenthood, whereas men report greater uncertainty regarding their parental identity than women.

The perinatal period, which extends from pregnancy through the first postpartum year, represents a critical phase during which parental identity becomes a central component of development [7]. During the transition to motherhood or fatherhood, identity must be restructured to incorporate the new caregiving role. However, this process is not automatic. It involves cognitive and emotional dynamics through which the previous sense of self assimilates representations of the child and the parent–child relationship, transforming a mental representation into a real and unique human relationship [3,4,8].

Research on parental identity during the perinatal period has largely focused on women, with early studies examining how the transition to motherhood reconfigures personal identity. According to Rubin [3], this process entails a complex network of biological, social, and psychological transformations that culminate in the consolidation of the maternal role. Rubin [3] proposed that pregnancy allows women to prepare psychologically for motherhood by incorporating the child into their self-system and life space through successive refinements of an ideal maternal identity. This process is not a single occurrence confined to the transition to first-time motherhood. Rather, it recurs with each subsequent pregnancy, as every childbearing experience requires the woman to reposition herself within her life space and self-system [3].

Mercer [8] reformulated maternal identity as the process of “becoming a mother”. This process involves “grief work,” requiring women to relinquish roles and lifestyles that are incompatible with the maternal role. Within this framework, maternal identity development is understood as inherently contextual: it unfolds alongside the child’s growth and is shaped by factors such as maternal health, previous caregiving experience, self-esteem, social support, and infant responsiveness. Consequently, validation from health professionals and experienced peers, along with positive feedback from the infant, can significantly strengthen maternal confidence [8].

Although research on parental identity during the perinatal period has predominantly focused on women, a growing body of research suggests that it is also relevant to understanding men’s experiences of parenthood and family functioning. Within symbolic interactionism, Stryker’s theory [9] conceptualizes identities as meanings attached to social positions and roles that individuals occupy. Thus, when individuals become parents, they assume a new social position—that of a parent—and develop a corresponding identity. Applied to fatherhood, identity theory distinguishes between fatherhood as a status of identity and the more specific role identities associated with fathering, such as caregiving, providing, and offering emotional support. This framework further distinguishes between identity salience, which refers to the relative importance of the father role compared with other roles, and identity centrality, which refers to the absolute importance that an individual attributes to being a father as part of his sense of self. This distinction is important because salience and centrality may predict paternal involvement differently. More recently, research has proposed a new model of paternal identity that links it to paternal self-efficacy and existing identity hierarchies, as well as to fathers’ experiences with their own parents, paternal role models, and the characteristics of their coparenting partners [10].

Although well-established theoretical frameworks exist [2,3,8], empirical research on parental identity during the perinatal period remains fragmented. Moreover, there is a lack of a rigorous organization and synthesis of empirical evidence on “parental identity” among both mothers and fathers during this period. Therefore, this study aimed to systematically identify, describe, and synthesize original evidence on the conceptualization, measurement, and associated factors of maternal and paternal identity during the perinatal period.

## 2. Materials and Methods

This study was reported in accordance with the Preferred Reporting Items for Systematic Reviews and Meta-Analyses (PRISMA) guidelines [11]. The completed PRISMA checklist is provided in the Supplementary Materials (Table S5). The systematic review protocol was prospectively registered in the International Prospective Register of Systematic Reviews (PROSPERO; registration number CRD420261376351). Several amendments were made to the registered protocol. Specifically, the risk-of-bias assessment was expanded to include the NOS-xs for cross-sectional studies in addition to the original NOS specified in the protocol.

### 2.1. Search Strategy and Eligibility Criteria

A comprehensive search of scientific literature was conducted across six databases: MEDLINE (via PubMed), EMBASE, PsycINFO, Scopus, Web of Science, and CINAHL. These databases were selected to provide a comprehensive coverage of the disciplinary perspectives from which parental identity has been studied: MEDLINE and EMBASE for biomedical and obstetric research; PsycINFO for psychological constructs such as identity and role transition; CINAHL for the nursing and midwifery literature, in which research on the maternal role is well represented; and Scopus and Web of Science as multidisciplinary databases that extend coverage to the social sciences and journals not indexed in the other sources. The final search of all databases was conducted in October 2026.

Eligibility criteria were defined according to the population (P), exposure (E), comparison (C), outcome (O), and study design (S) (PECO-S) framework. Studies were eligible for inclusion if they met the following criteria: (a) pregnant individuals and their partners during pregnancy and up to 12 months postpartum (perinatal period) (P); (b) the perinatal period, defined as pregnancy through the first year postpartum (I); (c) no comparison group was required (C); (d) parental identity, maternal identity, and/or paternal identity was included as a study variable (O); and (e) the study was an original quantitative investigation employing a cross-sectional, cohort, or case–control design (S). No publication date, language or country restrictions were applied in the search.

The exclusion criteria were as follows: studies involving families pursuing parenthood through adoption, foster care, or similar pathways; experimental studies, qualitative and mixed-methods studies; systematic reviews and meta-analyses; theoretical articles; editorials; and conference abstracts without complete data. Details of the search strategy are provided in the Supplementary Materials (Table S1).

### 2.2. Study Selection and Data Extraction

Three reviewers (ML, AC, and IC) conducted study selection and data extraction independently, working in pairs under the supervision of EM. First, duplicate records were removed. The remaining studies were then screened by title and abstract, after which the full-text articles were assessed for eligibility. The following information was extracted from each study: authors; year of publication and country; study design; sample characteristics, including parental gender, parity, age, target population, and sample size; perinatal stage (pregnancy or postpartum); conceptualization and definition of identity; measurement of identity, including the instrument used and its validation status; the role of identity in the analysis (e.g., predictor, outcome, primary outcome, correlate, or mediator); principal findings; associated variables; effect sizes and statistical results; and risk of bias.

Given the substantial conceptual and methodological heterogeneity across the included studies in identity constructs, measurement instruments, study designs, and perinatal stages, a meta-analysis was not considered appropriate. Instead, a narrative synthesis was conducted, grouping the studies according to their conceptualization of identity, measurement approaches, and associated variables. No data transformations or procedures for handling missing summary statistics were necessary, since the results were presented descriptively, as reported in the individual studies. Table 1 and Table 2 summarize the study characteristics, identity constructs, principal findings, associated variables, and key statistical results. No subgroup analyses, meta-regressions, or sensitivity analyses were conducted, and no pooled effect measures were calculated. The results are presented as reported in each study (*r*, β, OR, and mean differences).

**Table 1:** Characteristics of the included studies (*n* = 15).

Author (Year)	Study Design	Country	Sample (n; Parental Gender; Age; Population)	Perinatal Stage & Parity
[12]	Longitudinal	US	*n* = 11; women; 32.54 (±5.01); previously infertile and never infertile mothers	Pregnancy (36–40 gestational weeks) and postpartum (4–6 weeks); primiparous
[13]	Longitudinal	US	*n* = 421; women; 26.5 (±4.54); military sample	Pregnancy (first to third trimester) and postpartum (6 months); primiparous and multiparous
[14]	Cross-sectional	Australia	*n* = 115; men; 30.7; fathers	Pregnancy (first trimester); primiparous
[15]	Longitudinal	Australia	*n* = 193; men; 30.8 (±5.58); fathers	Pregnancy first and third trimesters); primiparous
[16]	Cross-sectional	US	*n* = 70; men; 24 years, range 20–42; fathers	Pregnancy (last month); primiparous
Cook et al. (2007) [17]	Longitudinal	US	*n* = 68; both parents; mothers: 24.45 (4.99), fathers: 26.73 (5.93); parents	Pregnancy (third trimester) and postpartum (3–6 months); primiparous
[18]	Cross-sectional	Poland	*n* = 150; both parents; 27.35 (±4.55); parents	Postpartum (6 months); primiparous
[19]	Longitudinal	Korea	*n* = 55; women; users: 29.40 (3.20), nonusers: 28.60 (3.30); mothers who used or did not use Sanhujoriwon centers	Postpartum (2–4 days, 3 weeks, 4–6 weeks, and 12–14 weeks); primiparous
[20]	Cross-sectional	Korea	*n* = 88; women; 33.91 (±3.61); pregnant women diagnosed with gestational diabetes mellitus	Pregnancy (24–33 gestational weeks); primiparous and multiparous
[21]	Cross-sectional	Korea	*n* = 196; women; 31 (±3.9); mothers	Postpartum (1–6 weeks); primiparous and multiparous
[22]	Cross-sectional	Italy	*n* = 319; women; 33.74 (±5.05); pregnant women	Pregnancy (24–33 gestational weeks); primiparous and multiparous
[23]	Cross-sectional	Korea	*n* = 333; women; 35.29 (±3.72); women with high-risk pregnancies	Pregnancy (20–37 gestational weeks); primiparous
[24]	Cross-sectional	Korea	*n* = 212; women; 32.74 (±3.65); mothers in a Sanhujoriwon center	Postpartum (2 weeks in a care center); primiparous
[25]	Longitudinal	US	*n* = 136; women; 26.00 (3.60); pregnant women	Pregnancy (24–40 gestational weeks) and postpartum (9–14 weeks); primiparous
[26]	Longitudinal	UK	*n* = 59; women; 31.79 (±5.79); women diagnosed with gestational diabetes mellitus	Pregnancy (1 gestational week and 1 gestational month); primiparous and multiparous

Note: Age is reported in years as mean (standard deviation), unless otherwise indicated.

### 2.3. Quality Appraisal and Risk of Bias

Study quality was assessed by three reviewers (ML, AC, IC), who worked in pairs. We used the Newcastle–Ottawa Scale (NOS) [27] to assess the quality of cohort and case–control studies and the Newcastle–Ottawa Scale–xs (NOS-xs) [28] to assess the quality of cross-sectional studies. Both instruments assess selection, comparability, and outcome assessment. To ensure a standardized evaluation across different study designs, risk-of-bias thresholds were aligned with established methodological literature. For cohort studies evaluated via the original NOS [12] (0–9 stars): low risk of bias (7–9 stars), moderate risk of bias (5–6 stars), and high risk of bias (≤4 stars). For cross-sectional analytical studies, thresholds were applied according to the validation framework of the NOS-xs tool [28], which maintains an equivalent 9-star rating system across three adapted domains: sample selection (max 2 stars); measurement of exposure/outcome (max 4 stars); and confounding control (max 3 stars). And studies were classified as having low (7–9 stars), moderate (4–6 stars), and high (0–3 stars) risk of bias.

No formal method was used to assess the risk of bias arising from missing results, as protocol registration information was unavailable for most of the included studies. Similarly, no formal assessment of the certainty of the evidence (e.g., GRADE) was conducted because the narrative nature of the synthesis and the substantial heterogeneity across studies limited its applicability.

## 3. Results

### 3.1. Selected Studies

**Figure 1:**
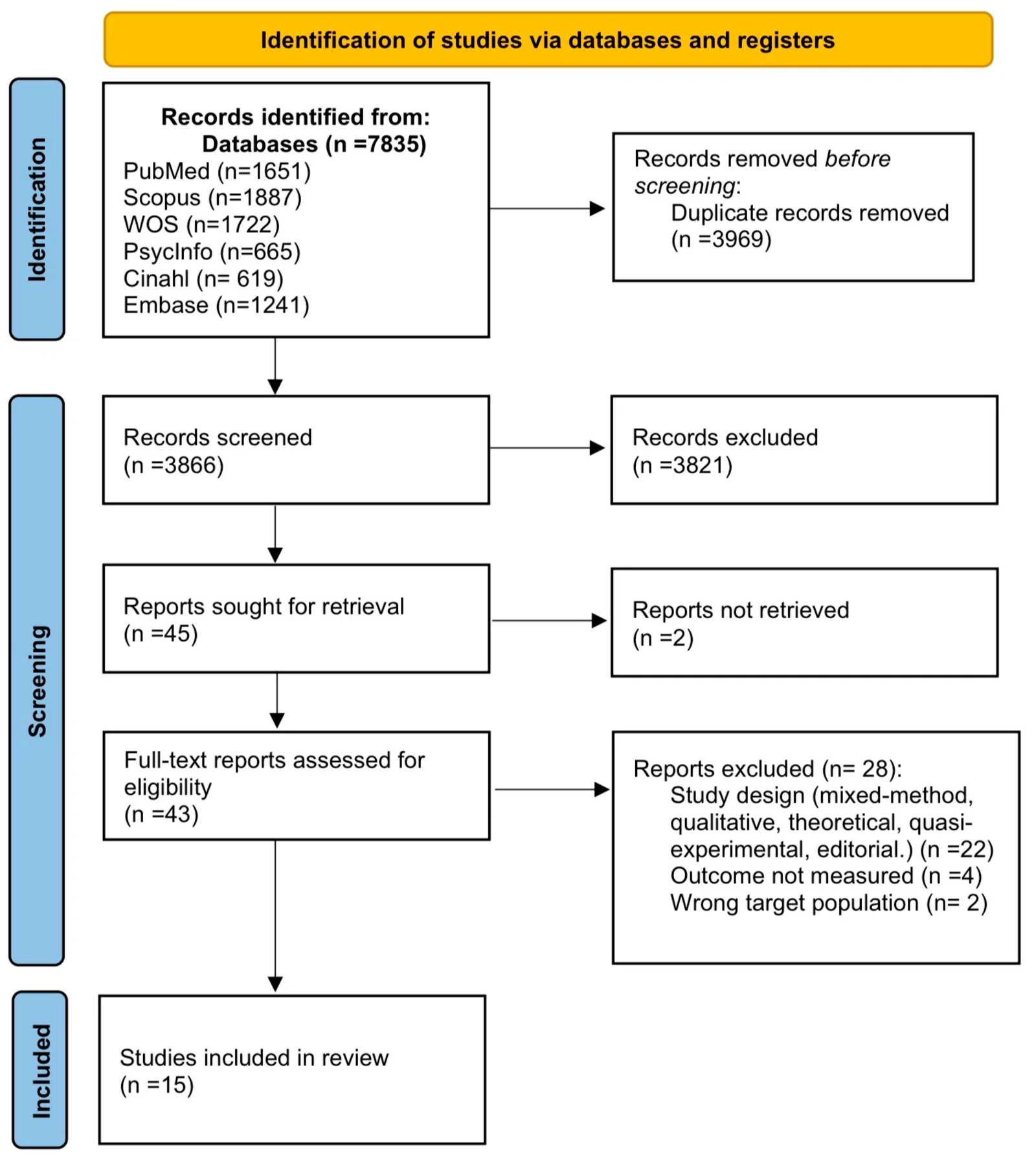
PRISMA 2020 flow diagram.

A total of 7835 records were initially identified through database searches. After removing 3969 duplicate records, the titles and abstracts of the remaining records were screened. Of these, 3821 were excluded, and two reports could not be retrieved. Subsequently, 43 full-text articles were assessed for eligibility. During full-text screening, 28 articles were excluded for the reasons reported in the Supplementary Materials (Table S2). Ultimately, 15 studies met the eligibility criteria and were included in the systematic review (Figure 1).

### 3.2. Study Characteristics

The characteristics of the 15 studies included in this systematic review are presented in Table 1, with additional information provided in the Supplementary Materials (Table S3). Collectively, the studies included 2426 participants, with individual sample sizes ranging from 11 [12] to 421 [13]. The review included data from six countries, mainly South Korea (*n* = 5), followed by the United States (*n* = 5) and Australia (*n* = 2). The remaining studies were conducted in Italy (*n* = 1), Poland (n = 1), and the United Kingdom (*n* = 1).

The included studies spanned nearly five decades, from 1979 to 2023. Notably, seven were published between 2019 and 2023, reflecting renewed scholarly interest in parental identity.

#### 3.2.1. Parental Gender

Out of the 15 included studies, ten exclusively focused on maternal identity, three on paternal identity [14,15,16] and two adopted a couple’s perspective by involving both parents [17,18]. Furthermore, the only couple study assessing postpartum identity found no significant gender differences in identity consolidation [18]. The primary correlates also vary by gender. Maternal identity constructs were associated with physical health, emotional clarity, and social support [19,20,21]. Paternal identity mostly aligned with relational variables such as couple relation quality, partner-related support or recognition of their role as caregivers [16,19,20].

#### 3.2.2. Perinatal Stage

Seven of the 15 included studies focused exclusively on pregnancy, four on the postpartum period, and four covered both stages. Postpartum assessments ranged from the first few days after childbirth to six months of postpartum, although most studies focused on the first six weeks.

#### 3.2.3. Study Design

The 15 included studies differed according to study design. Cross-sectional studies (*n* = 8) [14,16,18,20,21,22,23,24] provided valuable data on concurrent associations between parental identity, coping strategies, and attachment, though their design inherently precludes establishing temporal sequence or causality. In contrast, longitudinal and cohort studies (*n* = 7) [12,13,15,17,19,25,26] were essential for capturing temporal trajectories, identity stability, and role transition across perinatal stages (a detailed methodological appraisal and risk-of-bias evaluation for each design is presented in Section 3.4.)

### 3.3. Main Results

The principal findings of the 15 studies included in this systematic review are presented in Table 2. Additional study characteristics are provided in the Supplementary Materials (Table S4).

**Table 2:** Identity construct definitions, measurements, main findings, and associated variables.

Author	Identity Construct and Definition	Measurement	Principal Findings	Associated Variables
[12]	Maternal identity: “A developmental process culminating in a realization of the cognitive and affective attributes of the self as mother relative to the infant.” [3,9]	Walker’s Semantic Differential Scale—Myself as Mother and My Baby Scale by Walker [29,30]	Previously infertile mothers showed a weakening of maternal identity over the assessment period. By contrast, mothers with no history of infertility exhibited a strengthening of maternal identity.	Positive (+):no history of infertility; feeding behavior Negative (−): history of infertility.
[13]	Maternal identity attainment: A progressive process of self-assessment and development through which a woman transitions from being a woman without a child to a woman with a child while recognizing the child’s separateness [3,8,31]	Prenatal Self-Evaluation Questionnaire (PSEQ) [31]; Postpartum Self-Evaluation Questionnaire (PPSEQ) [32]	Family adaptability and received social support facilitated the formation of the maternal role and identity by reducing conflict during this process.	Positive (+): Consolidated identity before childbirth; social support. Negative (−): Husband’s military deployment.
[14]	Paternal identity/father status prominence: The subjective importance of fatherhood to the self [33,34]	The Pie (ad hoc adapted version) [35,36]	Father status prominence and role content were positively associated with prenatal paternal–fetal bonding. The caregiver and emotional-support roles were the strongest predictors.	Positive (+): father status prominence; caregiver role; emotional support for the mother. Non-significant (NS): Provider role content; Coach role content
[15]	Paternal identity: The process of self-reorganization that begins during pregnancy [33,34]	The Pie [35,36,37]	Paternal identity did not change significantly across pregnancy.	Positive (+): Paternal–fetal attachment; caregiver role content; emotional supporter role content. Non-significant (NS): Paternal identity set prominence over time.
[16]	Paternal identity: How a man perceives himself as a father during the prenatal period [38]	Ad hoc interview-based composite measure of paternal identity developed for the study [16]	Stronger prenatal paternal identity was associated with greater marital satisfaction, a shorter marriage, a more positive attitude toward pregnancy, and more previous contact with infants. Weaker paternal identity was associated with higher income and greater support from the fathers’ own parents.	Positive (+): Marital satisfaction; positive feelings about pregnancy; contact with infants. Negative (−): Income; duration of marriage before childbirth; current parental support.
Cook et al. (2007) [17]	Paternal identity style: The pattern through which individuals seek and process self-relevant information [37,39]	Identity Style Inventory (ISI) [39]	Fathers’ identity styles were associated with both marital closeness and perceived paternal involvement after childbirth. Fathers with a diffuse identity style reported the greatest marital closeness and self-reported paternal involvement, whereas mothers perceived fathers with a normative identity style as the most involved.	Positive (+): diffuse identity style; marital intimacy and father involvement. Negative (−): normative style Non-significant (NS): Informational identity style.
[18]	Identity processes: A specific domain of global identity that is not static but develops through dynamic processes of commitment and exploration that are constructed and readjusted over time [40,41]	Dimensions of Identity Development Scale (DIDS) [41]	No overall gender differences in identity processes were identified. Mothers of older infants reported greater commitment-making and identification with commitment, as well as less ruminative exploration.	Positive (+): satisfaction with life; positive affect; identification with commitment (IC); commitment-making (CM). Negative (−): Negative affect; ruminative exploration (ER). Non-significative (NS): Parents’ sex.
[19]	Maternal identity: A component of maternal adjustment during the postpartum transition [42]	Walker’s Semantic Differential Scale—Myself as Mother [30], adapted by Koh [43]	In both groups, maternal identity was significantly lower at 4–6 weeks postpartum than at 2–4 days and 12–14 weeks postpartum.	Positive (+): Use of a *sanhujori* facility. Negative (−): Postpartum fatigue; childcare stress.
[20]	Maternal identity: A pregnant woman’s internalization of the maternal role and her emotional and cognitive connection with her new identity and child [3,8]	Maternal Identity Scale developed by Kim and Hong [44]	Emotional clarity was positively associated with maternal identity and fetal attachment. Both emotional clarity and maternal identity significantly predicted fetal attachment.	Positive (+): emotional clarity; fetal attachment; religion; family history of gestational diabetes mellitus
[21]	Maternal identity: A mother’s perceptions and emotions regarding herself in relation to her child [45,46]	Maternal Identity Scale [43]	Maternal identity significantly predicted maternal sensitivity.	Positive (+): Social support; sensitivity; maternal–fetal attachment; employment status. Negative (−): Gestational age at birth.
[22]	Maternal identity as a “turning point.” Turning points are episodes that involve a substantial change in an individual’s life [47]	Centrality of the Event Scale (CES) [48]	Women who perceived pregnancy. Turning points are episodes that involve a substantial change in an individual’s life.	Positive (+): Prenatal attachment; posttraumatic stress disorder symptoms. Non-significant (NS): Parity; history of miscarriage; mode of conception.
[23]	Maternal identity: Women’s perceptions and feelings about themselves as mothers in relation to the mother–infant relationship that develops after childbirth [44]	Maternal Identity Scale developed by Kim and Hong [44]	Maternal identity was one of the five indicators of the adaptation mode. Women with high-risk pregnancies had lower maternal identity scores than women with low-risk pregnancies.	Positive (+): Adaptative coping strategies; marital adjustment; quality of life. Negative (−): Uncertainty; mental fatigue; antenatal depression; anxiety; maladaptive coping strategies.
[24]	Maternal identity: A cognitive–affective development process that forms part of the transition to motherhood and directly influences maternal well-being and satisfaction [49]	Walker’s Semantic Differential Scale—Myself as Mother [30], adapted by Koh [43]	Mothers with a stronger maternal identity tended to report greater satisfaction with their use of postpartum care centers.	Positive (+): Perceived health status; partnership with *Sanhujoriwon* staff; educational support system Negative (−): Childcare-related stress; postpartum depression. Non-significant (NS): Care center satisfaction.
[25]	Maternal Identity/Maternal Role Attainment: A construct encompassing behavioral, subjective, and affective dimensions [3,8]	Walker’s Semantic Differential Scale—Myself as Mother and My Baby Scale [50]	Prenatal maternal attachment was positively associated with all measures of maternal role attainment, whereas postpartum depression was negatively associated with these measures.	Positive (+): attachment; feeding; competence; infant care. Negative (−): Postpartum depressive symptoms.
[26]	Maternal identity salience: The perceived importance of being a “mother-to-be” during pregnancy [9]	Adapted version of The Pie [51]	Most participants regarded their identity as a “mother-to-be” as very important. The findings suggested that a diagnosis of gestational diabetes mellitus coincided with the adoption of a highly valued maternal identity.	Positive (+): risk perception; social support Non-significant (NS): Identity salience; affective response; self-efficacy.

#### 3.3.1. Measurement

The findings revealed substantial variation and methodological heterogeneity across the literature. Several studies used validated instruments specifically designed to assess maternal identity. These included Walker’s Semantic Differential Scale—Myself as Mother and My Baby Scale [30,50] (*n* = 2) [12,25] and its adaptation by Koh [43] (*n* = 2) [19,24]; the Maternal Identity Scale developed by Kim and Hong [44] (*n* = 2) [20, 23]; the Maternal Identity Scale developed by Koh [43] (*n* = 1) [21]; and the Prenatal Self-Evaluation Questionnaire [31] and Postpartum Self-Evaluation Questionnaire [32] (*n* = 1) [13].

Other studies assessed parental identity indirectly through related constructs. These measures included the Centrality of Event Scale [48] (*n* = 1) [22], which was used to assess the centrality of pregnancy within individuals’ life narratives; the Dimensions of Identity Development Scale [41] (*n* = 1) [18], which captures general identity processes; and the Identity Style Inventory [39] (n = 1) [17].

The literature on paternal identity was particularly fragmented. The measures used included visuospatial role-prioritization tasks such as The Pie [35,36,51] (*n* = 3) [14,15,26], and an ad-hoc composite measure of paternal identity derived from interviews (*n* = 1) [16].

#### 3.3.2. Conceptualization

The included studies (*n* = 15) drew on a wide range of definitions and theoretical frameworks to operationalize parental identity. Broadly, these frameworks fall into two principal theoretical dimensions: structural models, which view parental identity as a relatively stable configuration of traits or behavioral styles, and process-oriented approaches, which conceptualize it as a dynamic construction that evolves over time.

Studies adopting a structural framework sought to define patterns and styles of parental identity during the perinatal period and to identify their specific characteristics. Within this dimension, researchers drew extensively on Stryker’s symbolic interactionist identity theory [9] to examine how the salience of the parental role is organized within a dynamic hierarchy of social identities. This perspective was complemented by McCall and Simmons’s framework [33], which was used to assess status prominence and role content among fathers, and Berzonsky’s model [39], which classifies sociocognitive identity styles—such as informational, normative, and diffuse orientations—during the transition to parenthood.

In contrast, process-oriented studies sought to understand the development and consolidation of parental identity at different stages of the perinatal experience. This paradigm is historically grounded in Rubin’s theory of maternal identity [3], which defines becoming a mother as an active cognitive process of “binding-in” the child into the self-system, and Mercer’s “becoming a mother” framework [8], which describes a developmental trajectory from rigid adherence to the maternal role to the consolidation of a personal maternal identity.

Moreover, a critical analysis of the literature indicates that these studies evaluated constructs that, while conceptually interconnected, represent distinct psychological dimensions and are not interchangeable. To resolve this conceptual heterogeneity, the reviewed constructs were categorized into two main categories: direct and indirect constructs of parental identity.

Studies using direct constructs of parental identity assessed self-definition and adaptation to the parental role, targeting two distinct facets of the transition: a) internal identity consolidation (*n* = 7), which evaluates the intrapsychic, affective-cognitive integration of the self as a parent (maternal identity and paternal identity/image formation), capturing one’s self-definition as a caregiver [12,16,19,20,21,23,24]; and b) the behavioral role attainment constructs (*n* = 2), which evaluate functional competence, mastery of caregiving tasks, and behavioral adaptation (maternal role attainment). While role attainment is often treated synonymously with identity, it reflects behavioral performance rather than internal self-concept consolidation [13,25].

Studies focused on the use of indirect constructs of parental identity evaluated parental identity indirectly through broader identity frameworks: (a) studies evaluated role salience and status prominence (*n* = 3) [14,15,26] by assessing the relative rank or priority assigned to the parental role within an individual’s overall hierarchy of social identities; (b) event centrality (*n* = 1) [22] by measuring the extent to which pregnancy anchors the personal life narrative and serves as a central turning point via the Centrality of Event Scale; and (c) general identity processes and styles (*n* = 2) [17,18] by examining universal sociocognitive identity mechanics, such as commitment, exploration dimensions, or identity styles, applied directly to the perinatal transition.

Although direct constructs (internal identity and role attainment) and indirect constructs (role salience, event centrality, and general identity styles) capture distinct levels of experience and yield heterogeneous empirical findings, incorporating this broader spectrum is theoretically justified. The transition to parenthood may be conceptualized as a multi-faceted process involving both the potential acquisition of a specific parental self-concept and the broader structural reorganization of an individual’s social role hierarchy and life narrative. Evaluating both levels allows this review to explore how these varied identity dimensions are differentially consolidated, contextually prioritized, or experienced across diverse perinatal populations.

#### 3.3.3. Associated Factors

Maternal and paternal identity were generally studied independently. Only one study compared maternal and paternal identity during the perinatal period, specifically the postpartum period, and found no significant differences in identity processes between the two groups [18].

Longitudinal studies of mothers showed that maternal identity during pregnancy may predict maternal identity during the postpartum period (*n* = 2) [12,13]. Maternal identity declined between the first few days after childbirth and 4–6 weeks postpartum before beginning to stabilize (*n* = 1) [19]. By contrast, paternal identity remained stable across the trimesters of pregnancy due to the reorganization of social roles (*n* = 1) [14]. Prenatal maternal attachment and social support were also associated with postpartum maternal identity (*n* = 2) [13,25].

Cross-sectional studies identified positive associations between maternal identity during pregnancy and prenatal attachment (*n* = 5) [15,20,21,25], emotional clarity (*n* = 1) [20], and quality of life (*n* = 1) [23]. Surprisingly, Ionio et al. [22] also found that greater centrality of maternal identity within an individual’s overall identity was associated with posttraumatic stress disorder symptomatology, particularly intrusion and avoidance. Certain aspects of women’s reproductive histories and pregnancy-related circumstances were also associated with stronger maternal identity during pregnancy, including no history of infertility (*n* = 1) [12] and a diagnosis of gestational diabetes mellitus (*n* = 2) [20,26].

During the postpartum period, maternal identity was positively associated with satisfaction with support programs (*n* = 1) [24], life satisfaction (*n* = 1) [18], maternal sensitivity (*n* = 1) [21], satisfaction with feeding practices (*n* = 2) [12,25] and infant care [14,15,25]. Conversely, childcare-related stress (*n* = 2) [19,24] and the severity of depressive symptoms were negatively associated with postpartum maternal identity [19,25]. Across the reviewed literature, maternal age, parity, gestational age, birth weight, and sociodemographic factors—including education, employment, income, and religion—were also examined as correlates of maternal identity.

The empirical evidence indicates that parental identity development is not a static milestone confined to the birth of a first child. Rather, the birth of each subsequent child initiates a renewed process of active identity negotiation, expanding the dimensions of the parental role, and creating a unique relationship with each infant [23]. Even among multiparous parents, each transition represents a distinct adaptive challenge characterized by unique life-space dynamics and unresolved conflicts surrounding the imagined “ideal” parent [3].

In one study, diffuse paternal identity style during pregnancy was associated with greater marital intimacy and involvement in fatherhood during the postpartum period than an information-centered identity (*n* = 1) [17]. During the prenatal period, paternal identity was positively associated with prenatal attachment (*n* = 1) [14], marital satisfaction, and previous experience with infants (*n* = 1) [16], but negatively associated with higher income and greater perceived support from the father’s family (*n* = 1) [16].

A total of seven studies (*n* = 7) reported non-significant associations between parental identity and various sociodemographic, relational, or health outcomes. Specifically, no significant differences were identified among overall identity processes between mothers and fathers (*n* = 1) [18], and no changes in paternal identity prominence across pregnancy trimesters were found (*n* = 1) [15]. Likewise, specific role dimensions such as the breadwinner role (*n* = 1) [14] or an informational identity style (*n* = 1) [17] showed no significant association with paternal–fetal attachment or marital intimacy. Finally, regarding health and behavioral outcomes, identity salience did not significantly predict adherence to gestational diabetes management guidelines (*n* = 1) [26], maternal identity was not an independent predictor of satisfaction with postpartum care centers in multivariable analysis (*n* = 1) [24], and prior gynecological history (such as parity or history of miscarriage) showed no significant relationships with pregnancy event centrality in women identity (*n* = 1) [22].

### 3.4. Quality of the Studies

The eight cross-sectional studies generally demonstrated acceptable methodological quality, with seven classified as low risk-of-bias (scores of 7–8 stars) [20,21,22,23,24], two as moderate-risk (5–6 stars) [14,18], and one as high-risk (2 stars) [16]; limitations remained in adjusting for potential confounding factors and justification of sample size. By contrast, most of the seven cohort studies had important methodological limitations, with only one study achieving a low risk of bias (8 stars) [13], four showing moderate risk (5–6 stars) [15,19,25,26], and two demonstrating high risk (4 stars) [15,17] due to inadequate follow-up and limited sample representativeness, which affected the validity of their findings. Overall, only three studies were classified as having a high risk of bias [12,16,17]. In addition, some findings should be interpreted with caution because they were based on indirect measures or instruments requiring further validation (*n* = 8). The risk-of-bias assessments for each included study are presented in Appendix A (Table A1 and Table A2); further information on the measures is provided in Table 2.

The possibility of reporting bias cannot be ruled out. Studies with small samples reported significant findings [12], while studies reporting null or negative findings were notably absent from the databases searched.

No statistical synthesis was conducted because of substantial heterogeneity across the included studies in terms of identity constructs, measurement instruments, and study designs. Consequently, heterogeneity was not formally investigated, and no sensitivity analyses were performed. The findings are therefore presented narratively in Section 3.2 and Section 3.3. In addition, no formal assessment of the certainty of the overall body of evidence was conducted because of the narrative nature of the synthesis. The findings should therefore be interpreted cautiously, particularly given the predominance of cross-sectional designs and the methodological heterogeneity across studies.

## 4. Discussion

The findings of this review support both classical and contemporary theoretical frameworks, suggesting that parental identity is an evolving psychological construct that develops throughout the perinatal period. The progressive development of maternal identity observed during this period is consistent with maternal role attainment theory [18,21]. This process is characterized by the continuous cognitive–affective integration of the maternal identity during pregnancy, followed by a temporary decline at 4–6 weeks postpartum and subsequent stabilization [9,13]. Paternal identity, in turn, is consistent with symbolic interactionist perspectives. It appears to remain structurally stable across pregnancy trimesters of pregnancy while developing through a cognitive reorganization of social role hierarchies. Within this process, relational factors—particularly caregiver self-concepts and spousal support—are associated with paternal–fetal attachment and the quality of coparenting [10,11,51].

This systematic review showed that maternal and paternal perinatal identity continue to be treated as two independent constructs within separate lines of research. Only one study compared maternal and paternal identity processes and characteristics during this period [18]. This separation limits the development of a coherent conceptual framework for parental identity during the perinatal period and precludes firm conclusions regarding similarities and differences between maternal and paternal identity processes and patterns. Nevertheless, the included studies suggest that maternal and paternal characteristics, together with reciprocal support between partners, may shape each partner’s parental identity. Future research should therefore examine parental identity through direct comparisons between mothers and fathers while also considering potential patterns of coparental identity.

In addition, few studies have conducted robust longitudinal assessments of parental identity across different stages of the perinatal period [12,16,17,25]. Future studies should address this gap by assessing parental identity at multiple time points, from the first trimester of pregnancy to 2–3 years after childbirth.

Furthermore, the relationship between parental identity and parental mental health remains underexplored. The reviewed studies identified cross-sectional associations between a weaker maternal identity and more severe depressive symptoms during the postpartum period [19,25]. They also yielded an apparently counterintuitive finding: greater centrality of maternal identity during pregnancy was associated with more severe posttraumatic stress disorder symptoms [22]. Future research should more comprehensively examine the relationships between symptoms of perinatal mental health disorders and the characteristics of parental identity, including paternal identity.

From a methodological perspective, the risk-of-bias assessment highlights specific parameters that require optimization in future research. In cohort studies (*n* = 7) evaluated with the Newcastle–Ottawa Scale (NOS) [27], criteria related to exposure ascertainment and verifying the absence of the outcome at baseline were consistently satisfied, whereas sample representativeness, selection of non-exposed comparison groups, and adequacy of follow-up retention were met least frequently. In cross-sectional studies (*n* = 8) assessed via the adapted NOS-xs [28], exposure and outcome measurement criteria were widely fulfilled due to the use of structured instruments, but formal sample size justification and statistical adjustment for key confounding variables (e.g., parity, sociodemographic factors, and psychiatric history) were satisfied least frequently. To enhance methodological quality, future empirical studies should employ prospective cohort designs featuring representative sampling, a priori sample size calculations, longitudinal retention protocols to minimize attrition bias, and robust multivariable controls for confounding factors.

These distinct developmental processes might have important clinical implications for early intervention. Social support and emotional clarity appear to facilitate the development of a consolidated parental identity, whereas potential risk factors may operate through different pathways for mothers and fathers. A history of infertility has been associated with a less favorable maternal identity trajectory [12], whereas conditions requiring behavioral adaptation, such as gestational diabetes mellitus, may serve as turning points that increase the salience of maternal identity [20,26]. By contrast, factors such as perinatal depression and childcare-related stress may hinder the consolidation of the maternal role [24].

However, these findings must be interpreted in light of substantial conceptual and methodological limitations. The literature shows considerable measurement heterogeneity, with instruments ranging from validated scales, such as Walker’s Semantic Differential Scale and Kim and Hong’s Maternal Identity Scale, to nonstandardized ad hoc or single-item measures [31,39]. Small sample sizes in some studies [12] further limit the robustness and generalizability of the findings. In addition, the geographic concentration of studies in South Korea and the United States, the exclusion of qualitative research, and potential publication bias favoring positive findings warrant caution. Without forgetting the three studies qualified as high-risk in the bias assessment [12,16,17]. Establishing standardized, gender-sensitive definitions and robust measurement instruments is essential to accurately evaluate parental identity as a potential protective factor in perinatal mental health.

This review contributes to a structured overview of how parental identity has been conceptualized, measured, and studied during the perinatal period, and highlights several priorities for future research. A shared conceptual framework and validated, comparable instruments are needed, particularly for fathers, whose identity has been assessed with heterogeneous and partly ad hoc measures. Prospective longitudinal designs would help clarify how identity develops and how it relates to psychological outcomes, since more than half of the included studies were cross-sectional. Dyadic designs and samples including multiparous parents are also needed, as only one study compared mothers and fathers [18] and most focused on primiparous parents. Only then could the clinical utility of assessing parental identity be evaluated.

While exploring parental identity during perinatal consultations represents a promising hypothesis for identifying vulnerable families, the current observational evidence does not establish the diagnostic utility or clinical effectiveness of routine identity screening. Rather than an established clinical mandate, identity-based assessment should be viewed as a prospective framework for future implementation research, aiming to test whether brief clinical evaluation of identity processes can reliably detect families in need of targeted psychosocial support.

## 5. Conclusions

Future research should seek to establish a theoretical consensus that defines parental identity clearly and consistently. Greater standardization of measurement approaches and the development of instruments capable of capturing the complexity of identity across various contexts and populations are also needed.

## Data Availability

No new data were created or analyzed in this study. Data sharing is not applicable to this article.

## Supplementary Materials

The following supporting information can be downloaded at https://www.mdpi.com/article/10.3390/healthcare14213809/s1, Table S1: Complete search strategies for all electronic databases; Table S2: Full-text studies excluded from the systematic review; Table S3: Complete descriptives of included studies; Table S4: Complete identity construct definitions, main findings and associated variables; Table S5: PRISMA checklist 2020.

## Abbreviations

The following abbreviations are used in this manuscript:

GDM	Gestational Diabetes Mellitus
NOS	Newcastle–Ottawa Scale
NOS-xs	Newcastle–Ottawa Scale adapted for cross-sectional studies
PTSD	Posttraumatic stress disorder

## Appendix A

**Table A1:** Risk-of-bias assessment of cohort studies using the Newcastle–Ottawa Scale (NOS) (*n* = 7).

Author (Year)	S1	S2	S3	S4	Comparability	O1	O2	O3	Total	Risk of Bias
[12]			★	★		★	★		4	High
[13]	★	★	★	★	★★	★	★		8	Low
[15]	★	★	★	★		★	★		6	Moderate
Cook et al. (2007) [17]			★	★		★	★		4	High
[19]		★	★	★		★	★		5	Moderate
[25]	★	★	★	★		★	★		6	Moderate
[26]		★	★	★	★		★		5	Moderate

Note: S1—representativeness of the exposed cohort; S2—selection of the nonexposed cohort; S3—ascertainment of exposure; S4—demonstration that the outcome of interest was not present at the beginning of the study; comparability—comparability of cohorts based on the design or analysis; O1—assessment of outcome; O2—whether the follow-up period was sufficiently long for outcomes to occur; O3—adequacy of follow-up. Each star (★) represents one point awarded for fulfilling a specific methodological quality criterion of the Newcastle–Ottawa Scale (NOS) for cohort studies (maximum total score = 9 stars: Selection max 4★, Comparability max 2★, Outcome max 3★) [27].

**Table A2:** Risk-of-bias assessment of cross-sectional studies using the Newcastle–Ottawa Scale adapted for cross-sectional studies (NOS-xs) (*n* = 8).

Author (Year)	I1	I2	I3	I4	I5	I6	Total	Risk of Bias
Habib et al. (2006) [14]	★ (b)		★	★★	★	★	6	Moderate
[16]	★ (b)		★				2	High
[18]	★ (b)		★★	★★			5	Moderate
[20]	★ (b)	★	★★	★★	★	★	8	Low
[21]	★ (b)	★	★★	★★	★	★	8	Low
[22]	★ (b)		★	★★	★★	★	7	Low
[23]	★ (b)	★	★★	★	★	★	8	Low
[24]	★ (b)	★	★★	★	★	★	7	Low

Note: I1—representativeness of the sample; I2—sample size; I3—assessment of exposure(s); I4—assessment of outcome(s); I5—adjustment for confounder(s); I6—assessment of confounder(s). The maximum scores are 2 stars for study-sample selection (I1–I2), 4 stars for the assessment of exposures and outcomes (I3–I4), and 3 stars for confounding factors (I5–I6). Each star (★) indicates one point awarded for satisfying a specific quality criterion in the NOS-xs scale (maximum total score = 9 stars). For Item I1 (representativeness), ★ (b) denotes 1 star awarded for non-randomized samples considered ‘somewhat representative’ of the target population [28].
