# Supplementary material for: Maternal and Paternal Identity During the Perinatal Period: A Systematic Review of Conceptualization, Measurement, and Associated Factors
Source: Healthcare (Basel). 2026 Nov 7;14(21):3809. doi: 10.3390/healthcare14213809 (PMC13762606; doi:10.3390/healthcare14213809)
Supplement: Supplementary file 1 [file healthcare-14-03809-s001.zip › healthcare-4633994-supplementary.pdf]

Table S1: Complete search strategies for all electronic databases.

Web Survey	Filters applied	Search Strategies	Date of Search	Results (n= 6.401)
PubMed	-	("Pregnancy"[Mesh] OR "Postpartum Period"[Mesh] OR "Perinatal Care"[Mesh] OR pregnan*[Title/Abstract] OR prenatal*[Title/Abstract] OR antenatal*[Title/Abstract] OR perinatal*[Title/Abstract] OR postnatal*[Title/Abstract] OR postpartum*[Title/Abstract] OR puerper*[Title/Abstract]) AND (motherhood[Title/Abstract] OR fatherhood[Title/Abstract] OR mother*[Title/Abstract] OR father*[Title/Abstract] OR maternal[Title/Abstract] OR paternal[Title/Abstract] OR parent*[Title/Abstract]) AND (identity[Title/Abstract] OR identities[Title/Abstract] OR "parental identity"[Title/Abstract] OR "maternal identity"[Title/Abstract] OR "paternal identity"[Title/Abstract] OR "role identity"[Title/Abstract])	28/10/2026	1.651
Scopus	Article	(TITLE-ABS-KEY (pregnancy) OR TITLE-ABS-KEY (postpartum period) OR TITLE-ABS-KEY (perinatal care) OR TITLE-ABS-KEY (pregnant) OR TITLE-ABS-KEY (prenatal) OR TITLE-ABS-KEY (antenatal) OR TITLE-ABS-KEY (perinatal) OR TITLE-ABS-KEY (postnatal) OR TITLE-ABS-KEY (postpartum) OR TITLE-ABS-KEY (puerper)) AND (TITLE-ABS-KEY (motherhood) OR TITLE-ABS-KEY (fatherhood) OR TITLE-ABS-KEY (mother) OR TITLE-ABS-KEY (father) OR TITLE-ABS-KEY (maternal) OR TITLE-ABS-KEY (paternal) OR TITLE-ABS-KEY (parent)) AND (TITLE-ABS-KEY (identity) OR TITLE-ABS-KEY (identities) TITLE-ABS-KEY (parental identity) OR TITLE-ABS-KEY (maternal identity) OR TITLE-ABS-KEY (paternal identity) OR TITLE-ABS-KEY (role identity)) AND (LIMIT-TO (DOCTYPE , "ar"))	28/10/2026	1.887
Web Of Science	Article	((TS= (Pregnancy OR Pregnant OR Postpartum period OR Perinatal Care OR Perinatal OR Prenatal OR Postnatal OR Antenatal OR Postpartum OR Puerper)) AND (TS=(Motherhood OR Fatherhood OR Father OR Mother OR Parental OR Paternal OR Maternal OR Parent)) AND (TS=(Identity OR Identities OR Parental identity OR Paternal Identity OR Maternal identity OR Role Identity)))	28/10/2026	1.722
PsychInfo	Journal Article	Tiab(pregnancy OR pregnant OR postpartum period OR perinatal care OR prenatal OR antenatal OR postpartum OR postnatal OR perinatal) AND tiab(motherhood OR fatherhood OR father OR mother OR maternal OR paternal OR parental OR parent) AND tiab(identity OR identities OR parental identity	28/10/2026	665

		OR maternal identity OR paternal identity OR role identity)		
Cinahl	Academic Publication	XB (Pregnancy OR Postpartum period OR Pregnant OR Perinatal care OR Prenatal OR Antenatal OR Postnatal OR Perinatal OR Postpartum OR Puerper) AND XB (Motherhood OR Fatherhood OR Mother OR Father OR Maternal OR Paternal OR Parental OR Parent) AND XB (Identity OR Identities OR Parental identity OR Maternal identity OR Paternal identity OR Role identity)	29/10/2026	619
Embase	Article	('pregnancy':ti,ab,kw OR 'puerperium':ti,ab,kw OR 'perinatal care':ti,ab,kw OR pregnant:ti,ab,kwOR 'prenatal':ti,ab,kw OR antenatal:ti,ab,kw OR perinatal:ti,ab,kw OR postnatal:ti,ab,kw OR 'postpartum':ti,ab,kw OR puerper:ti,ab,kw) AND ('mother':ti,ab,kw OR 'father':ti,ab,kw OR 'maternal':ti,ab,kw OR paternal:ti,ab,kw OR 'parent':ti,ab,kw) AND ('identity':ti,ab,kw OR identities:ti,ab,kw OR 'paternal identity':ti,ab,kw OR 'role identity':ti,ab,kw OR 'maternal identity':ti,ab,kw) NOT (review:it OR 'systematic review':it OR 'meta analysis':it)	28/10/2026	1.241

Table S2. Full-text studies excluded from the systematic review (n = 28).

**Author, year**	Exclusion Criteria
Berlincioni et al., 2015	Study design is qualitative
Bleich JD, 1991	Study design is qualitative
Bouche-Florin et al., 2017	Study design is qualitative
Cabrera, J., 2018	Study design is theoretical
Cassidy et al., 2008	Study design is quasi-experimental
Chae et al., 2011	Study design is quasi-experimental
Chea et al., 2011	Study design is quasi-experimental
Fasanghari et al., 2019	Study design is a clinical trial
García-Romero et al., 2026	Study design is mixed-method
Howarth et al., 2011	Study design is qualitative
Kim et al., 2018	Study design is quasi-experimental
Liddelow et al., 2023	Population not exclusive to the perinatal period
Marsiglio, W, 2008	Study design is an editorial
Messias et al., 2007	Study design is qualitative
Moon et al., 2015	Outcome not measured
Navarro et al., 2025	Study design is qualitative
Naziri et al., 2006	Study design is qualitative
Newby et al., 2021	Outcome not measured

Olivares et al., 2024	Outcome not measured and wrong concept of identity
Oliveira et al., 2021	Study design is qualitative
Ollivier et al., 2024	Study design is qualitative
Özkan et al., 2011	Study design is quasi-experimental
Porter et al., 2020	Study design is qualitative
Ribeiro et al., 2017	Study design is qualitative
Shin et al., 2004	Outcome not measured
Spinelli et al., 2016	Study design is qualitative
Walsh et al., 2023	Study design is qualitative
Yamasaki et al., 2023	Population not exclusive to the perinatal period

Table S3. Complete Descriptives of Included Studies (n= 15)

Author (year)	Study design	Country	Sample	Perinatal stage & Parity	Identity Measure
[12]	Descriptive pilotlongitudinal study	US	n=11<br>Sex: Woman.<br>*Age*: 32.54 (± 5.01)<br>*Population*: Previously infertile and never infertilemothers.	Pregnancy (36-40 gw) and postpartum (4-6 pw)<br>Primiparous	Semantic Differential Scale - Myself as Mother and My Baby Scale by Walker [29,30]
[13]	Longitudinal study	US	n=421<br>*Sex*: Women<br>*Age*: 26.5 (±4.54)<br>*Population*: Military sample	Pregnancy (3 trimesters) and postpartum (6 months)<br>Primiparous and multiparous.	Prenatal Self-Evaluation Questionnaire (PSEQ) [31]; Postpartum Self-Evaluation Questionnaire (PPSEQ) [32]
[14]	Cross-sectionalcorrelationalstudy	Australia	n=115<br>*Sex*: Men<br>*Age*: 30.7<br>Population: Fathers	Pregnancy (first trimester)<br>Primiparous	The Pie (ad hoc adapted version) [35,36]
[15]	Longitudinal observationalstudy	Australia	n=193<br>*Sex*: Men<br>*Age*: 30.8 (±5.58)<br>*Population*: Fathers	Pregnancy (1st -3^{rd}trimester)<br>Primiparous	The Pie [35,36,37]

[16]	Cross-sectional study	US	n=70<br>*Sex*: Men<br>*Age*: 24, range 20-42<br>*Population*: Fathers;	Pregnancy (last month)<br>Primiparous	Interview-based composite father identity ad-hoc measure. The measure was ad-hoc created for this study. [16]
[17]	Longitudinal survey study	US	n= 68<br>*Sex*: Women and men<br>*Age*: Mothers: 24.45 (± 4.99); Fathers: 26.73 (± 5.93)<br>*Population*: Parents	Pregnancy (last trimester) and postpartum (3-6 months)<br>Primiparous	Identity Style Inventory (ISI) [39]
[18]	Cross-sectionalstudy	Poland	n=150<br>*Sex*: Women and men<br>*Age*: 27.35 (±4.55)<br>*Population*: Parents	Postpartum (6 months)<br>Primiparous	Dimensions of Identity Development Scale (DIDS) [41]
[19]	Longitudinal descriptive study	Korea	n=55<br>*Sex*: Women<br>*Age*: Use group: 29.4 (±3.2); non-use group: 28.6 (±3.3)<br>*Population*: Mothers using/not using Sanhujoriwons	Postpartum (2-4 days; 3 pw; 4-6 pw; 12-14 pw)<br>Primiparous	Semantic Differential Scale - Myself as Mother by Walker [30], adapted by Koh [43]

[20]	Cross-sectional correlational	Korea	n=88<br>*Sex*: Women.<br>Age: 33.91 (± 3.61)<br>*Population*: Pregnant womendiagnosed with Gestational Diabetes Mellitus.	Pregnancy (24-33 gw)<br>Primiparous and multiparous.	Maternal Identity Scale developed by Kim and Hong [44]
[21]	Cross-sectionaldescriptive study	Korea	n=196<br>Sex: Women<br>Age: 31 (± 3.9)<br>Population: Mothers	Postpartum (1-6 pw)<br>Primiparous and multiparous.	Maternal Identity Scale [43]
[22]	Cross-sectionalstudy	Italy	n=319<br>*Sex*: Women<br>*Age:* 33.74 (±5.05)<br>*Population*:Pregnant women	Pregnancy (3rd trimester)<br>Primiparous and multiparous.	Centrality of the Event Scale (CES) [48]
[23]	Cross-sectional	Korea	n=333<br>*Sex*: Women.<br>*Age*: 35.29 (±3.72)<br>*Population*: High-risk pregnant woman.	Pregnancy (20-37 gw)<br>Primiparous	Maternal Identity Scale by Kim and Hong [44]
[24]	Descriptive and correlational study with a cross-sectional design	Korea	n=212<br>*Sex*: Women.<br>*Age*: 32.74 (± 3.65)<br>*Population*: Mothers in Sanhujoriwon center	Postpartum (2 week in care center)<br>Primiparous	Semantic Differential Scale - Myself as Mother by Walker [30], adapted by Koh [43]

[25]	Longitudinal study	US	n=136<br>*Sex*: Women.<br>*Age*: 26 (± 3.6)<br>*Population*: Pregnant woman.	Pregnancy (24-40 gw) and postpartum (9-14 pw)<br>Primiparous	Semantic Differential Scale - Myself as Mother and My Baby Scale by Walker [50]
[26]	Observationalsurvey study	UK	n=59<br>*Sex*: Women<br>*Age*: 31.79 (± 5.79)<br>*Population*: Women diagnosed with GDM	Pregnancy (1 week-1 month)<br>Primiparous and multiparous.	Adapted version of The Pie [51]

Note: gw= gestational weeks; pw= postpartum weeks; GDM= Gestational Diabetes Mellitus

Table S4: Complete identity construct definitions, main findings and associated variables.

Author	Identity Construct & Definition	Role in Analysis	Main Findings	Associated variables	Main Key Statistics
[12]	Maternal identity: "A developmental process culminating in a realization of the cognitive and affective attributes of the self as mother relative to the infant." [3,9]	Main outcome	Never infertile showed a strengthening in maternal identity across the testing period, resulting in a highly positive maternal self-evaluation and infant evaluation at four to six weeks postpartum. Mothers who were previously infertile showed a weakening in maternal identity across the testing period, resulting in lower maternal self-evaluations and infant evaluations.	Positive (+) = Never infertility status; feeding behavior. Negative (-) = Previous infertile status.	Postpartum SD-Self group difference U=0.00, p<.01; SD-Baby U=1.00, p<.01; change in SD-Self from prenatal to postpartum F(1,9) = 8.76, p<.01; SD-Self at postpartum correlated with maternal feeding behavior in previously infertile women r_s=.96, p<.01.
[13]	Maternal identity formation/attainment: Progressive self-assessment and developmental step from woman without child to woman with child, recognizing separateness of child. [3,8,31]	Main outcome and predictor	The family's adaptability and social support received function as facilitatingfactors that reduce conflict in the formation of the maternal role and identity. Conversely, the spouse's military deployment during the first trimester of pregnancy was identified as a risk factor that increases conflict in accepting the pregnancy.	Positive (+) = Solid identity before childbirth; social support. Negative (-) = husband's military deployment.	Prenatal n=421 (388 analyzed), postpartum n=113 military women. IDMORO predicts CMRT/SIC (sig.); SSI slope sig. CMRT; FADAPT borderline SIC; deployment sig. SIC.
[14]	Father status prominence/Father identity: Within identity	Predictor	Father status prominence and father status content were positively related to paternal-fetal bonding. Caregiver and emotional	Positive (+) = Father status prominence;	Mean father prominence: 20.8% (SD 14.0). PAAS: Global Attachment M=60.99 SD=7.32; Quality M=34.34 SD=3.51; Time in

	theory [33], father status prominence is the subjective importance of fatherhood to the self; status content refers to the meanings attached to being a father.[34]		supporter content were the strongest predictors of bonding; breadwinner, play/coach, and helper contents were not significant.	caregiver; emotional support of the mother. Non-significant (NS)= Provider role content; Coach role content.	Attachment Mode: M=18.31 SD=3.88. Regression: Caregiver content β=.45, p<.001 and Emotional Supporter content β=.26, p<.05 explained 36.5% of Global Attachment variance; Caregiver β=.43, p<.001 and Emotional Supporter β=.24, p<.01 explained 28.9% of Quality; Father Prominence β=.25, p<.01 and Caregiver β=.39, p<.001 explained 29.3% of Time in Attachment Mode.
[15]	Father identity: The process of self-reorganization that begins during pregnancy, where the man begins to see himself in relation to his unborn child, laying the foundations for the future father-child relationship [33,34]	Predictor and correlate	Father's identity did not change significantly across pregnancy.	Positive (+) = Paternal-fetal attachment; Caregiver role content; Emotional supporter role content. Non-significant (NS)= Paternal identity set prominence over time.	There was no significant change in either the kind of statuses that comprised the men's identity or in their fatherstatus prominence (paired sample t = 1.03, df = 67, p = 0.15, 1-tailed). In fact, this was the case for all other statuses. Father status prominence was not significantly different at trimester 3 (21.2%) from trimester 1 (20.8%)
[16]	Father identity: how a man thinks of himself as a father in the prenatal	Main outcome	Prenatal father identity varied widely across men, but it tended to be stronger when fathers reported higher marital satisfaction, a	Positive (+) = Marital satisfaction; affect about	Father's identity composite: mean=14, median=14, SD=2.9, observed range 7-20. Significant linear correlations with father

	period, reflecting the emotional capacity and readiness for parenting and forming the foundation of the future father-child relationship. [38]		shorter marriage before the first child, more positive feelings about the pregnancy, and more prior contact with infants. Father identity was lower among men with higher income and more support from their own parents.	pregnancy; contact with infants. Negative (-) = income; duration of marriage before childbirth; current parental support. Not lineal (U form) = closeness with own father and adequacy of father in meeting needs	identity: income r=-.32, p=.01; marital satisfaction r=.29, p=.02; duration of marriage before childbirth r=-.42, p=.001; affect about pregnancy r=.31, p=.01; contact with infants r=.27, p=.05; current parental support r=-.28, p=.05. Significant non-linear associations: closeness with own father F=4.0, p=.01; adequacy of father in meeting support needs F=9.70, p=.001
[17]	Paternal identity style is conceptualized following Erikson [37] and Berzonsky [39] as the pattern by which individuals seek and process self-relevant information	Predictor	Fathers' identity style was associated with both marital intimacy and perceived father involvement after birth. Diffuse fathers reported the highest marital intimacy and higher self-reported father involvement than information-oriented fathers, whereasmothers perceived normative fathers as the most involved. The study focuses on paternal identity rather than maternal identity.	Positive (+) = Diffuse style; marital intimacy and father involvement. Negative (-) = Normative style. Non-significant (NS)= Informational identity style.	Fathers: 28 were information-oriented (32%), 22 normative (25%), and 38 diffuse (43%). Identity styles significantly impactedall variables (p < .05). Fathers' marital intimacy: (F (2,84) = 6.78). Mothers' intimacy reports: (F (2,83) = 4.42). Fathers' self-reported involvement:(F (2,59) = 4.82). Mothers' reports of involvement: (F (2,57) = 3.41$).
[18]	Identity processes: It is a specific domain within the	Outcome	No sex differences in identity processes overall; mothers of older infants showed	Positive (+) = Satisfaction with	In mothers, older child age was associated with higher commitment making (t=2.537, p=.013),

	global identity that is not static, but a dynamic process of commitment and exploration that is built and readjusted over time, especially during the first months after the birth of the first child. [40,41]		higher commitment making and identification with commitment, and lower ruminative exploration. Satisfaction with life was linked to emotions and identity processes.	life; Ppositive affect; identification with commitment (IC), commitment making (CM), age of a child. Negative (-) = Ruminative exploration (ER); Negative affect. Non-significative (NS)= Parents' sex	higher identification with commitment (t=2.676, p=.009), and lower ruminative exploration (t=2.246, p=.028).
[19]	Maternal identity: is part of maternal adjustment during postpartum transition, following Mercer framework.[42]	Main outcome and correlate	Maternal identity was significantly lower at 4-6 weeks postpartum compared to 2-4 days and 12-14 weeks across both groups. Nonsignificant differences between Sanhujori facility users and non-users, but overall patterns showed a dip in maternal identity during the 4–6-week period.	Positive (+) = Sanhujori facility uses. Negative (-) = Postpartum fatigue; Childcare stress.	Maternal identity means: Use group T1(2-4d) M=54.67 SD=10.20, T2(4-6w) M=52.81 SD=10.66, T3(12-14w) M=57.14 SD=11.38; non-use T1 M=54.44 SD=9.33, T2 M=53.68 SD=8.04, T3 M=57.59 SD=7.68. Repeated measures ANOVA: Group F=0.03 p=.868, Time F=5.89 p=.004, Group×Time F=0.10 p=.875. Post-hoc: T1, T2 < T3 (p<.017).

[20]	Maternal Identity: Internalization of the role and the emotional and cognitive connection of the pregnant woman with her new identity and with her child. [3,8]	Mediator (between emotional clarity and fetal attachment) and predictor (of fetal attachment	Emotional clarity was positively related to maternal identity and fetal attachment. It affected maternal identity with 21.9% of explained variance. The emotional clarity and the maternal identity were significant predictors of fetal attachment by 57.7% of explained variance	Positive (+) = Emotional clarity; fetal attachment; religion; family history of Gestational Diabetes Mellitus.	Maternal identity mean = 127.82 ± 18.05. Correlation with emotional clarity: r=.430, p=.001. Correlation with fetal attachment: r=.766, p=.001; Maternal identity predicted fetal attachment, β=.765, p=.001,
[21]	Maternal identity: is the perception and emotion as a mother, seeing herself in relation to her child [45,46]. It can be achieved when a woman sees herself in relation to her child [46].	Predictor	Maternal identity was a significant predictor of maternal sensitivity; self-identity as a mother and identification with her baby were both associated with higher sensitivity.	Positive (+) = Social support; sensitivity; maternal-fetal attachment; employment status. Negative (-) = Gestational age at birth.	Mean of maternal identity: Self-identity as a mother (M=50.87, SD=7.59); Identification with her baby (M=33.69, SD=5.19)
[22]	Maternal/Personal identity: Maternal identity as: “turning points.” Those episodes that involve a substantial change in an individual’s life. [47]	Predictor	These results indicatedthat women that perceived pregnancy as a crucial phase for their life story and identity reported a higher level of prenatal attachment. Multiparous, younger, and lower-educated women scored higher on pregnancy centrality.	Positive (+) = Prenatal Attachment; PTSD symptoms. Non-significant (NS): Parity;	The CES explained 15% of variance in prenatal attachment (R2=.15, p<.001). CES scores correlated with PTSD symptomatology (r=.190, p=.011), especially intrusion and avoidance.

				History of miscarriage; Mode of conception.	
[23]	Maternal identity: Women's perceptionsand feelings as mothers about the mother-infant relationship that forms after childbirth through the physiological transition to motherhood and is the process of adapting to maternal behaviors and achieving the maternal role. [44]	Main outcome	Maternal identity formed one of the five indicators of the adaptation mode, which had a significant direct effect on quality of life; high-risk pregnant women had a mean maternal identity score of 126.51 ± 16.35, lower than values reported for low-risk pregnant women in cited comparison studies	Positive (+) = Adaptative coping strategies; marital adjustment; quality of life. Negative (-) = Uncertainty; mental fatigue; antenatal depression; anxiety; maladaptive coping strategies.	Final model: adaptation mode b=.81, p=.034, directly affected QoL; maladaptive coping β=.46, p=.043, directly affected QoL; uncertainty had indirect effect β=.21, p=.004; adaptive coping indirect effect β=.36, p=.026;
[24]	Maternal identity: no formal definition was provided, but they understand maternal identity not as something static, but as a cognitive-affective development process that is part of the transition to motherhood and that directly influences	Predictor and correlate	Maternal identity was positively correlated with satisfaction with Sanhujoriwons, indicating that mothers with a stronger maternal identity tended to report greater satisfaction with postpartum care centreuse. However, maternal identity did not remain a significant independent predictor in the final hierarchical regression model once perceived health status,partnership with staff, and education support system were entered.	Positive (+) = Perceived health status; partnership with Sanhujoriwon staff; education support system. Negative (-) = Childcare stress and postpartum depression.	Maternal identity mean = 52.82 ± 8.16. Correlation with satisfaction with Sanhujoriwons: r = .26, p < .001. In hierarchical regression, maternal identity was not a significant predictor in Model II (B = 0.08, β = .06, t = 0.93, p = .353), Model III (B = -0.02, β= -.02, t = -0.28, p = .778), or final Model IV (B = 0.01, β = .01, t = -0.03, p = .973).

	the well-being and satisfaction of the mother. [49]			Non-significant (NS)= Care center satisfaction	
[25]	Maternal Identity/Maternal Role Attainment: involves not only the behavioral and subjective aspects of maternal role attainment but encompasses affective dimensions as well. [3,8]	Main outcome	Prenatal maternal attachment was positively related to all maternal role attainment measures; postpartum depression was negatively related to all maternal role attainment measures; prenatal attachment was not significantly related to postpartum depression.	Positive (+) = attachment; feeding; competence; infant care. Negative (-) = Postpartum depressive symptoms.	The prenatal attachment had M = 130.8 (SD = 13.1), the EPDS M = 6.8 (SD = 4.1), the Myself as Mother scale M = 64.8 (SD = 6.8), My Baby M = 37.5 (SD = 3.8) and Perceived Competence M = 5.07 (SD = 0.57)
[26]	Maternal identity salience is the perceived importance of being a "mother to be" during pregnancy, examined as part of the teachable moment model of behavior change. [9]	Predictor and outcome	Most participants (49 of 50) reported their identity as 'mother to be' as either very important or quite important. Resultsregarding identity highlights that diagnosis of GDM coincides with the adoption of a highly valued maternal identity. Several authors have suggested that adoption of this identity qualifies pregnancy as an important 'Teachable moment'	Positive (+) = Risk perception, social support. Non-significant (NS): Identity salience; affective response; self-efficacy.	Identity salience was very high. In regression, identity in univariate analysis (B= .21, p=.37), in model 1 (B=-0.02, p=.94), in model 2 (B=0.03, p=.92). Positive correlation (nonsignificant): identity salience + affective response (B=.21, p=.36), identity salience + risk perception (B=.01, p=.937)

Table S5. PRISMA checklist 2020 [11]

Section and Topic	Items #	Checklist item	Reported on page #
TITLE			
Title	1	Identify the report as a systematic review.	1
ABSTRACT			
Abstract	2	Provide a structured summary, including, as applicable: background; objectives; study eligibility criteria; data sources; participants and interventions; study appraisal and synthesis methods; results; limitations; conclusions and implications of key findings; systematic review registration number.	1-2
INTRODUCTION			
Rationale	3	Describe the rationale for the review in the context of existing knowledge	3
Objective	4	Provide an explicit statement of the objective(s) or question(s) the review addresses	3
METHODS			
Eligibility criteria	5	Specify the inclusion and exclusion criteria for the review and how studies were grouped for the syntheses	4
Information sources	6	Specify all databases, registers, websites, organizations, reference lists and other sources searched or consulted to identify studies. Specify the date when each source was last searched or consulted	3
Search strategy	7	Present the full search strategies for all databases, registers and websites, including any filters and limits used.	4
Selection process	8	Specify the methods used to decide whether a study met the inclusion criteria of the review, including how many reviewers screened each record and each report retrieved, whether they worked independently, and if applicable, details of automation tools used in the process.	4
Data collection process	9	Specify the methods used to collect data from reports, including how many reviewers collected data from each report, whether they worked independently, any	4

		processes for obtaining or confirming data from study investigators, and if applicable, details of automation tools used in the process.	
Data items	10a	List and define all outcomes for which data were sought. Specify whether all results that were compatible with each outcome domain in each study were sought (e.g., for all measures, time points, analyses), and if not, the methods used to decide which results to collect.	4
	10b	List and define all other variables for which data were sought (e.g.,participant and intervention characteristics, funding sources). Describe any assumptions made about any missing or unclear information.	4
Study risk of bias assessment	11	Specify the methods used to assess risk of bias in the included studies, including details of the tool(s) used, how many reviewers assessed each study and whether they worked independently, and if applicable, details of automation tools used in the process.	5
Effect measures	12	Specify for each outcome the effect measure(s) (e.g., risk ratio, mean difference) used in the synthesis or presentation of results.	4
Synthesis methods	13a	Describe the processes used to decide which studies were eligible for each synthesis (e.g., tabulating the study intervention characteristics and comparing against the planned groups for each synthesis (item #5)).	4
	13b	Describe any methods required to prepare the data for presentation or synthesis, such as handling of missing summary statistics, or data conversions	4
	13c	Describe any methods used to tabulate or visually display results of individual studies and syntheses.	4
	13d	Describe any methods used to synthesize results and provide a rationale for the choice(s). If meta-analysis was performed, describe the model(s), method(s) to identify the presence and extent of statistical heterogeneity, and software package(s) used	4

	13e	Describe any methods used to explore possible causes of heterogeneity among study results (e.g., subgroup analysis, meta-regression).	4
	13f	Describe any sensitivity analyses conducted to assess robustness of the synthesized result.	4
Reporting bias assessment	14	Describe any methods used to assess risk of bias due to missing results in a synthesis (arising from reporting biases).	5
Certainty assessment	15	Describe any methods used to assess certainty (or confidence) in the body of evidence for an outcome	5
RESULTS			
Study selection	16a	Describe the results of the search and selection process, from the number of records identified in the search to the number of studies included in the review, ideally using a flow diagram.	6
	16b	Cite studies that might appear to meet the inclusion criteria, but which were excluded, and explain why they were excluded.	6
Study characteristics	17	Cite each included study and present its characteristics.	4-5 (Table 1)
Risk of bias in studies	18	Present assessments of risk of bias for each included study	15-16 (Table A1; Table A2)
Results of individual studies	19	For all outcomes, present, for each study: (a) summary statistics for each group (where appropriate) and (b) an effect estimate and its precision (e.g. confidence/credible interval), ideally using structured tables or plots.	7-10 (Table 2)
Result of synthesis	20a	For each synthesis, briefly summarize the characteristics and risk of bias among contributing studies.	6-13
	20b	Present results of all statistical syntheses conducted. If meta-analysis was done, present for each the summary estimate and its precision (e.g., confidence/credible interval) and measures of statistical heterogeneity. If comparing groups, describe the direction of the effect.	13

	20c	Present results of all investigations of possible causes of heterogeneity among study results.	13
	20d	Present results of all sensitivity analyses conducted to assess the robustness of the synthesized results	13
Reporting biases	21	Present assessments of risk of bias due to missing results (arising from reporting biases) for each synthesis assessed.	13
Certainty of evidence	22	Present assessments of certainty (or confidence) in the body of evidence for each outcome assessed.	13
DISCUSSION			
Discussion	23a	Provide a general interpretation of the results in the context of other evidence.	13
	23b	Discuss any limitations of the evidence included in the review	14
	23c	Discuss any limitations of the review processes used	14
	23d	Discuss implications of the results for practice, policy, and future research.	14-15
OTHER INFORMATION			
Registration and protocol	24a	Provide registration information for the review, including register name and registration number, or state that the review was not registered.	3
	24b	Indicate where the review protocol can be accessed, or state that a protocol was not prepared.	3
	24c	Describe and explain any amendments to information provided at registration or in the protocol	3
Support	25	Describe sources of financial or non-financial support for the review, and the role of the funders or sponsors in the review	15
Competing interests	26	Declare any competing interests of review authors.	15
Availability of data, code, and other materials	27	Report which of the following are publicly available and where they can be found: template data collection forms; data extracted from included studies; data used for all analyses; analytic code; any other materials used in the review	15
